# Delayed Hemothorax Due to Rupture of a Right Inferior Phrenic Artery Pseudoaneurysm 67 Days After Rib Fractures: A Case Report

**DOI:** 10.7759/cureus.98612

**Published:** 2025-12-07

**Authors:** Akito Kubota, Momoko Sugiyama, Yasumasa Ode, Kenji Iwata, Asako Matsushima

**Affiliations:** 1 Department of Emergency and Critical Care, Nagoya City University, Graduate School of Medical Sciences, Nagoya, JPN; 2 Department of Diagnostic Radiology, Nagoya City University East Medical Center, Nagoya, JPN

**Keywords:** delayed hemothorax, inferior phrenic artery, rib fractures, thoracic injuries, traumatic pseudoaneurysm

## Abstract

Delayed hemothorax after blunt chest trauma usually develops within seven days, and the inferior phrenic artery (IPA) is a rare culprit vessel. We report a case of massive hemothorax that occurred 67 days after rib fractures due to rupture of an IPA pseudoaneurysm. A 73-year-old man was transported in shock with a five-day history of right hypochondrial pain. Non-contrast CT revealed a right pleural effusion with attenuation consistent with blood and multiple fractures of the right 8th-12th ribs, while contrast-enhanced CT demonstrated contrast extravasation adjacent to the right 10th rib. Angiography identified a pseudoaneurysm with active extravasation in a distal dorsal branch of the right IPA, and transcatheter arterial embolization was performed with successful hemostasis. He was admitted to the intensive care unit (ICU) and transfused; after stabilization of his hemodynamics, he was discharged from the ICU on hospital day 2. Percutaneous drainage was subsequently performed for the residual hematoma, and he was transferred to a rehabilitation hospital on hospital day 28. During the course, it was clarified that the multiple right rib fractures had been sustained in a fall 72 days before presentation; therefore, the hemothorax was diagnosed as a delayed hemothorax due to rupture of a right IPA pseudoaneurysm. We experienced a case of delayed hemothorax that developed on day 67 after rib fractures. In delayed hemothorax, the medical history should be traced back several months. Although rare, an IPA pseudoaneurysm can be a life-threatening cause of post-traumatic delayed hemothorax; therefore, in patients with lower rib fractures, an active diagnostic workup is essential.

## Introduction

With the rapid aging of Japan's population, traumatic injuries in older adults have become increasingly common [1], and the incidence of blunt thoracic trauma from falls has risen sharply [2]. Delayed hemothorax is a recognized complication of blunt chest trauma, occurring in approximately 12% of cases [3]. Prior reports indicate that 87% of delayed hemothoraces develop within seven days of injury and 54% within 48 hours [3]; the longest reported interval to onset is 60 days [4]. Intercostal arteries are most frequently implicated as the bleeding source in traumatic hemothorax [5]. In contrast, hemothorax attributable to the inferior phrenic artery (IPA) accounts for only 1.3% of cases yet has been associated with a mortality rate as high as 11% [6]. Here, we report a case of delayed hemothorax due to rupture of an IPA pseudoaneurysm presenting more than two months after the initial injury.

## Case presentation

A 73-year-old man presented with acute right-sided chest and upper abdominal pain and dyspnea that had progressively worsened over five days after he twisted his torso, 67 days after sustaining right posterolateral fractures of the 8th-12th ribs in a fall. On arrival, he was hemodynamically unstable with hypotension (blood pressure 79/54 mmHg) and tachycardia (heart rate 121 beats/min). Breath sounds were decreased over the right lower hemithorax, with chest wall tenderness along the right lower ribs. The venous blood gas analysis showed a pH of 7.360, pCO₂ of 43.1 mmHg, HCO₃⁻ of 23.7 mmol/L, and a base excess of −1.2 mmol/L, indicating no acid-base disturbance. The lactate level was mildly elevated at 3.3 mmol/L, raising suspicion of mild circulatory insufficiency. The hemoglobin level was 11.6 g/dL, indicating mild anemia. No abnormalities were observed in the coagulation and fibrinolytic systems.

**Figure 1:**
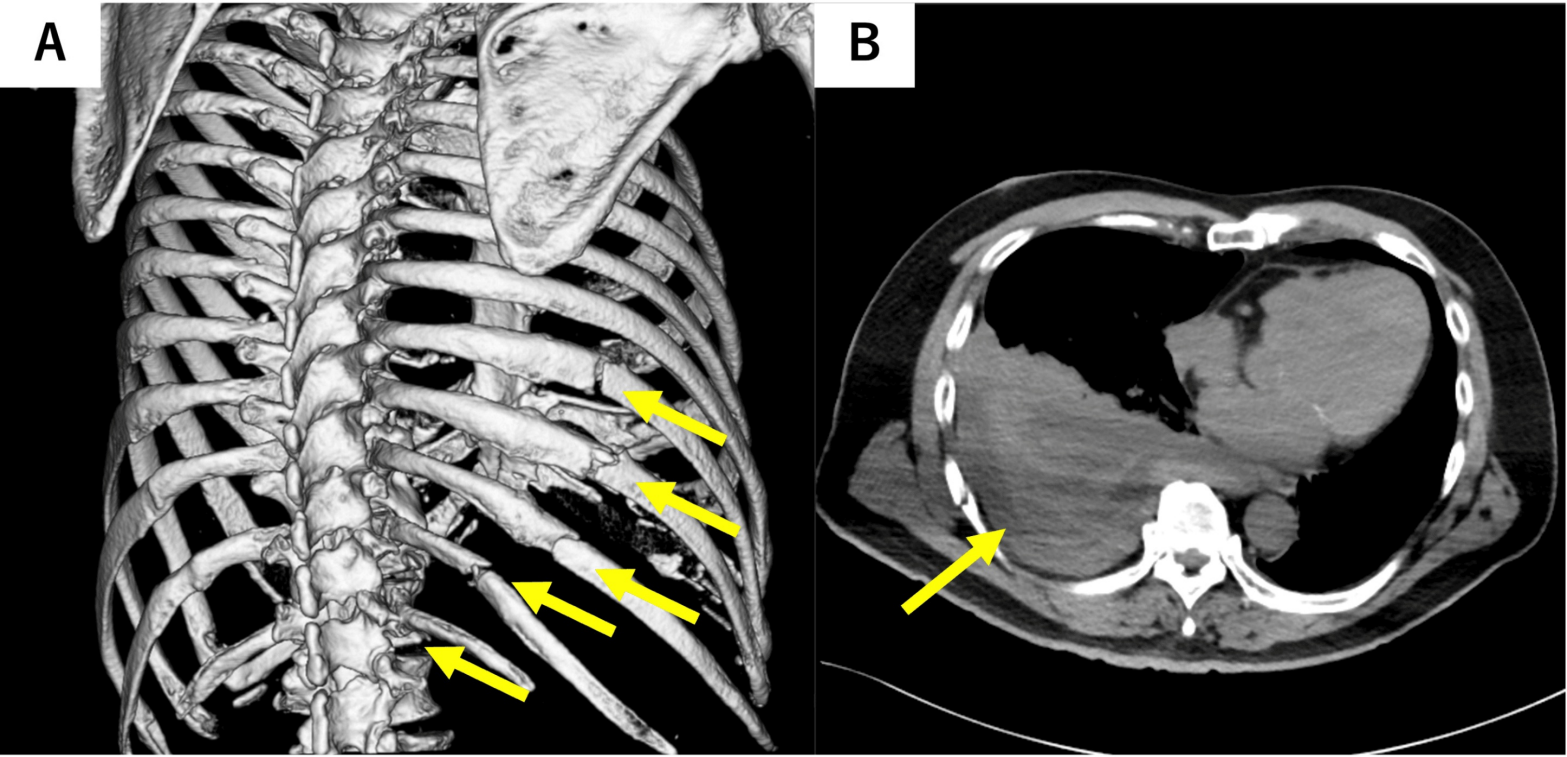
Rib fractures and hemothorax. (A) Three-dimensional CT bone reconstruction showing multiple healing fractures of the right 8th-12th ribs (arrows). (B) Axial non-contrast CT showing a large right pleural effusion compatible with hemothorax (arrow).

**Figure 2:**
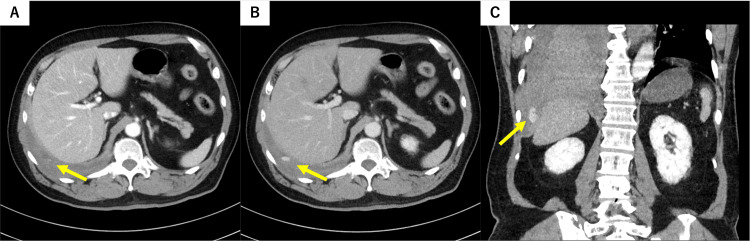
Contrast-enhanced CT identifying a pseudoaneurysm near the diaphragm. (A) Arterial-phase axial image showing a focal contrast blush along the right diaphragmatic surface (arrow). (B) Portal venous-phase axial image with persistent enhancement (arrow). (C) Coronal reformatted image demonstrating a saccular lesion abutting the parietal pleura near the right 10th rib (arrow), consistent with a pseudoaneurysm.

Non-contrast chest computed tomography (CT) showed multiple fractures of the right 8th-12th ribs (Figure 1A) and massive right hemothorax (Figure 1B). Contrast-enhanced CT demonstrated focal contrast extravasation along the right diaphragmatic surface near the right 10th rib (Figure 2A, arterial-phase axial; Figure 2B, portal venous-phase axial), and a coronal reformatted image delineated a saccular lesion abutting the parietal pleura, compatible with a pseudoaneurysm (Figure 2C). No findings suggestive of hepatic parenchymal or intestinal injury were observed.

**Figure 3:**
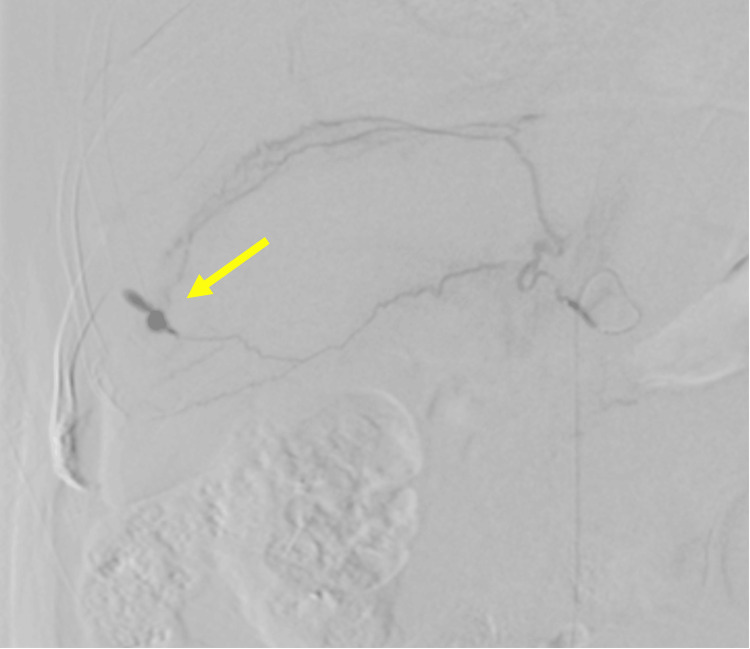
Angiography confirming the culprit lesion. Selective angiogram of the right inferior phrenic artery demonstrating a distal branch pseudoaneurysm with contrast extravasation (arrow) prior to embolization.

Given the ongoing bleeding, urgent angiography was performed. Digital subtraction angiography (DSA) via a femoral approach identified a pseudoaneurysm with active extravasation arising from a distal branch of the right IPA (Figure 3). Transcatheter arterial embolization (TAE) using n-butyl cyanoacrylate (NBCA) mixed with iodized oil (1:3) was performed, resulting in immediate occlusion of the pseudoaneurysm and cessation of extravasation. A thoracic drain was inserted for hemothorax evacuation. Postoperatively, the patient demonstrated rapid improvement in respiratory and hemodynamic status, with normalization of lactate. He was transferred out of the intensive care unit on postoperative day 2 and was later transferred to a rehabilitation hospital on postoperative day 28. No recurrence of bleeding was observed during follow-up.

## Discussion

We experienced a case in which a patient presented with delayed hemothorax 72 days after sustaining chest trauma due to a fall. The patient had complained of right hypochondrial pain for five days before presentation; if we assume bleeding began at that time, this represents a delayed hemothorax occurring 67 days after the initial rib fractures.

There is currently no unified definition of delayed hemothorax. Shorr et al. defined hemothorax appearing more than 24 hours after injury as "delayed" [7], whereas Ritter et al. defined it as hemothorax newly appearing more than two hours after the initial imaging study, even if that initial study was normal [8]. In our case, the event occurred 67 days after the initial injury, exceeding the longest interval of 60 days reported in previous studies [4]. When limiting the injured vessel to the IPA, the onset was 45 days later than that reported previously [6]. Delayed hemothorax typically presents with sudden dyspnea and chest pain. Prior studies identify advanced age (≥70 years), mid-to-lower rib injuries, and multiple rib fractures (≥3) as risk factors [9]. Our case met these symptoms and risk profiles. Therefore, in high-risk patients with otherwise unexplained hemothorax, regardless of the time elapsed since injury, vascular injury related to rib fractures or the formation of a pseudoaneurysm should be suspected.

Proposed mechanisms for delayed hemothorax after blunt chest trauma include chronic irritation from displaced rib fragments that injure vessels or the diaphragm and formation or rupture of a pseudoaneurysm [4,6,10]. Pseudoaneurysms following blunt chest trauma reflect arterial wall defects with extravascular blood accumulation; they have been reported to account for 12.5% of cases requiring TAE [11]. After hematoma formation, central liquefaction and residual communication with the parent artery create a cavity with turbulent flow that may enlarge and rupture unpredictably [12,13]. Potential mechanisms proposed for rupture of an enlarging pseudoaneurysm include repetitive mechanical irritation by rib-fracture fragments during respiration [10], secondary displacement of fracture fragments with changes in posture [14], and abrupt increases in intrathoracic pressure, such as with coughing or straining [15].

In our patient, fracture lines were present along the right anterolateral 8th-12th ribs, and the bleeding focus was identified approximately 2 cm from the right 10th rib. Selective DSA at this site demonstrated a pseudoaneurysm. As a mechanism of rupture, repetitive irritation by fracture fragments during routine respiration appeared unlikely, given that the pseudoaneurysm was not in close apposition to the chest wall. Moreover, prior reports implicating abrupt increases in intrathoracic pressure have largely involved more central vascular injuries, often accompanied by hemoptysis, which was not consistent with our case. Because right upper quadrant pain developed immediately after the patient twisted his torso five days before presentation and subsequently worsened, we considered two plausible explanations: (1) a post-traumatic pseudoaneurysm formed after the initial injury and then ruptured five days earlier because of secondary displacement of a fracture fragment, or (2) the torso twist caused a new vascular injury with pseudoaneurysm formation, resulting in intermittent bleeding and delayed hemothorax.

In prior reports of traumatic hemothorax requiring urgent TAE, the most common culprit vessels are the intercostal arteries (50%) and the internal mammary (internal thoracic) arteries (29.5%) [11]. Data on mortality are limited, but mortality associated with intercostal artery injury is reported at 9.1% (including cases with associated injuries) [16], and internal thoracic artery injury at 10.3% [17]. Hemothorax due to the IPA, as in our case, is rare, accounting for only 1.3% of cases, yet a mortality rate of 11% has been reported [6], suggesting severity comparable to hemothorax from intercostal or internal thoracic artery injuries.

Loukas et al. examined 330 adult cadavers [18] and detailed the origins of the IPA and its eight branches. On the right, the IPA originated directly from the celiac trunk in 40%, from the aorta in 38%, and from the renal artery in 17% of cases; nearly all eight branches supplied the ventral diaphragm. On the right IPA, cranially directed branches are described mainly as the branch to the inferior vena cava (IVC branch) and the diaphragmatic hiatus branch, arising from ventral divisions; these course deeper from the body surface than intercostal arteries and are therefore less susceptible to mechanical irritation by rib fragments. This anatomical relationship may help explain why the IPA is an uncommon culprit vessel in delayed hemothorax. In our case, bleeding arose from a distal dorsal branch of the right IPA; neither the IVC branch nor the hiatus branch was the culprit, raising the possibility of an anomalous branch traversing the diaphragm. It is important to note that, although rare, an IPA branch extending from the diaphragm into the thoracic cavity can be a cause of hemothorax.

## Conclusions

Rupture of a right IPA pseudoaneurysm can cause very late delayed hemothorax after rib fractures. Clinicians should maintain a high index of suspicion in patients with persistent or recurrent chest or upper abdominal pain, anemia, or an enlarging pleural effusion after such injuries, and obtain contrast-enhanced imaging to evaluate for active bleeding or pseudoaneurysm. Timely recognition of this entity and awareness of the IPA as a potential culprit vessel can expedite diagnosis, guide management, and help prevent life-threatening deterioration.
